# Ultrasonic radiomics in predicting pathologic type for thyroid cancer: a preliminary study using radiomics features for predicting medullary thyroid carcinoma

**DOI:** 10.3389/fendo.2025.1428888

**Published:** 2025-02-19

**Authors:** Dai Zhang, Fan Yang, Wenjing Hou, Ying Wang, Jiali Mu, Hailing Wang, Xi Wei

**Affiliations:** ^1^ Department of Diagnostic and Therapeutic Ultrasonography, Tianjin Medical University Cancer Institute and Hospital, National Clinical Research Center for Cancer, Tianjin, China; ^2^ Tianjin’s Clinical Research Center for Cancer, Tianjin Medical University Cancer Institute & Hospital, Tianjin, China; ^3^ State Key Laboratory of Druggability Evaluation and Systematic Translational Medicine, Tianjin Medical University Cancer Institute & Hospital, Tianjin, China; ^4^ Key Laboratory of Cancer Prevention and Therapy, Tianjin Medical University Cancer Institute & Hospital, Tianjin, China

**Keywords:** ultrasound, medullary thyroid carcinoma, papillary thyroid carcinoma, radiomics, personalized medicine

## Abstract

**Introduction:**

Medullary thyroid carcinoma (MTC) is aggressive and difficult to distinguish from papillary thyroid carcinoma (PTC) using traditional ultrasound. Objective to establish a standard-based ultrasound imaging model for preoperative differentiation of MTC from PTC.

**Methods:**

A retrospective study was conducted on the case data of 213 thyroid cancer patients (82 MTC, 90 lesions; 131 PTC, 135 lesions) from the Department of Diagnostic and Therapeutic Ultrasonography, Tianjin Medical University Cancer Institute and Hospital. We constructed clinical model, radiomics model and comprehensive model by executing machine learning algorithms based on baseline clinical, pathological characteristics and ultrasound image data, respectively.

**Results:**

The study showed that the comprehensive model observed the highest diagnostic efficacy in differentiating MTC from PTC with AUC, sensitivity, specificity, positive predictive value, negative predictive value and accuracy of 0.93, 0.88, 0.82, 0.77, 0.91, 85.8%. Delong test results showed that the comprehensive model was significantly better than the clinical model (Z=-3.791, P<0.001) and the radiomics model (Z=-2.017, *P*=0.044). Calibration curves indicated the comprehensive model and the radiomics model exhibited better stability than the clinical model. Decision curves analysis (DCA) demonstrated that the comprehensive model had the highest clinical net benefit.

**Discussions:**

Radiomics model is effective in identifying MTC and PTC preoperatively, and the comprehensive model is better. This approach can aid in identifying the pathologic types of thyroid nodule before clinical operation, supporting personalized medicine in the decision-making process.

## 1 Introduction

Thyroid cancer has been reported to account for the 9th highest incidence of all malignant tumors worldwide (1). Among these cases, the majority are papillary thyroid carcinomas (PTCs) (2). Although PTC is generally a low-risk tumor with a good prognosis, some patients may choose to undergo active surveillance. However, this approach is not suitable for patients with medullary thyroid carcinomas (MTC). MTC originates from parafollicular C cells. It is an aggressive thyroid malignancy with neuroendocrine features, accounting for 1-2% of all thyroid cancers but contributing to 8-13% of thyroid cancer-related deaths (3, 4).

Ultrasound is a valuable tool for detecting and diagnosing thyroid disorders, particularly thyroid nodules. The American College of Radiology (ACR) 2017 edition of the TI-RADS grading system (2017-ACR-TI-RADS) has been widely recognized for its ability to predict malignancy in thyroid nodules, though most studies have primarily focused on PTC (5–10). Some organizations have found that the ACR TI-RADS classification remains valid for malignant risk management in MTC, but it is less sensitive and less accurate than for PTC (11, 12). Therefore, it is crucial for clinicians and patients to differentiate MTC from PTC to facilitate early diagnosis and treatment of MTC, reduce mortality, and minimize unnecessary surgical interventions.

Radiomics refers to the high dimension, automated extraction of quantitative features from medical images that are not recognizable to the human eye, mining high-dimensional data to capture intra-tumor heterogeneity (13). Studies have been published on the application of radiomics in differentiating benign and malignant thyroid nodules and preoperatively predicting cervical lymph node metastasis in thyroid cancers (14, 15). However, studies on the preoperative differentiation of MTC from PTC based on radiomics are rarely reported. Therefore, this study aims to construct a clinical model, a radiomics model, and a comprehensive model to identify MTC and PTC preoperatively and to investigate the clinical value of these models.

## 2 Materials and methods

### 2.1 Study population

Clinical and ultrasound imaging data of patients diagnosed with MTC and PTC, confirmed by surgical pathology, were retrospectively collected from June 2018 to June 2022. The MTC cases were consecutively selected, while the clinical data of patients with PTC during the same period were randomly chosen.

Inclusion criteria were as follows: (1) Patients who underwent routine ultrasound examination within 1 week before surgery, with standardized image retention, relevant imaging data, and diagnostic results available. (2) Patients who visited our hospital and underwent initial thyroid surgery. (3) Complete postoperative pathology and immunohistochemistry results were available. (4) No history of other tumors.

Exclusion criteria were defined as follows: (1) Poor quality of ultrasound images affecting image segmentation and feature extraction. (2) Patients who had undergone interventional treatments, including radiofrequency ablation or microwave intervention, or had isotope radiotherapy and radiotherapy to the head and neck, prior to the examination. (3) Patients with incomplete clinical information.

**Figure 1:**
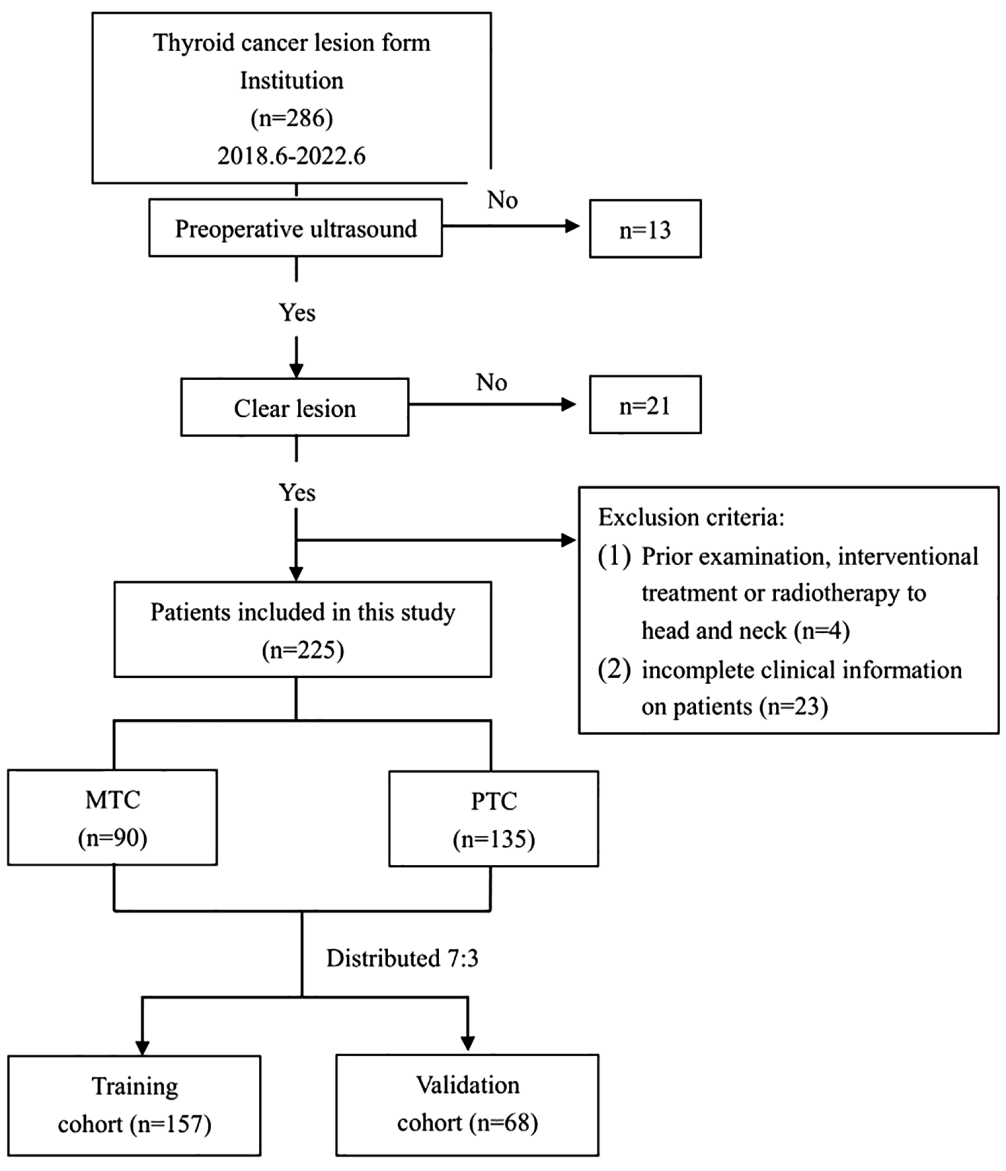
Project flowchart.

A total of 225 lesions from 213 eligible patients were ultimately included in the study. The flowchart of this study is shown in **Figure 1**. Among these, 82 patients had MTC, with a total of 90 lesions, consisting of 41 males and 41 females, with a mean age of 47.04 ± 12.94 years. Additionally, there were 131 patients with PTC, comprising 135 lesions, including 29 males and 102 females, with a mean age of 41.9 ± 11.68 years.

The Ethics Committee approved this study, and the requirement for patients’ informed consent was waived.

### 2.2 Instruments and methods

#### 2.2.1 Ultrasonographic methods and image analysis

A Toshiba Aplio500, Philips EPIQ5 and Philips IU22 diagnostic ultrasound equipment were utilized during the examinations. The corresponding probe models used were PVT-375BT, C5-1, and C5-1, with frequency ranges of 2.5 to 7.0 MHz, 1.0 to 5.0 MHz, and 1.0 to 5.0 MHz, respectively. The patient was placed in the supine position, and the head was tilted to fully expose the location of the thyroid gland. A single 2D image of the largest cross-section of the tumor was retained.

Basic clinical information of the patients was recorded, including gender, age, and clinical symptoms, such as neck mass, neck discomfort, and hoarseness. Furthermore, a comprehensive record was maintained detailing the patient’s family history concerning thyroid cancer, their past history of Hashimoto’s thyroiditis (HT), along with the occurrence of extrathyroidal extension (ETE), lymph node metastasis, and the specific TNM staging of the tumor. Ultrasound imaging findings were documented and evaluated by three radiologists with 5-10 years of experience in thyroid ultrasonography. The findings included the location, size, composition, margin, echogenicity, aspect ratio, calcification, and blood flow. Among these factors, blood flow assessment includes intra-lesional blood flow testing (CDFI), vascular distribution, and the Adler grading system. Vascular distribution: Type I: no blood flow detected; Type II: blood flow detected within the nodule; Type III: blood flow detected at the periphery of the nodule; Type IV: blood flow detected both within and at the periphery of the nodule (16). Blood flow grading was assessed using the Adler semiquantitative method as follows (17): Grade 0 indicates no blood flow in the lesion; Grade 1 shows 1-2 punctate or rod-shaped vessels in the lesion; Grade 2 shows 3-4 punctate vessels or a distinct vessel that may be close to or longer than the radius of the lesion; Grade 3 displays more than 5 punctate vessels or two longer vessels in the lesion.

#### 2.2.2 Image segmentation and data pre-processing

**Figure 2:**
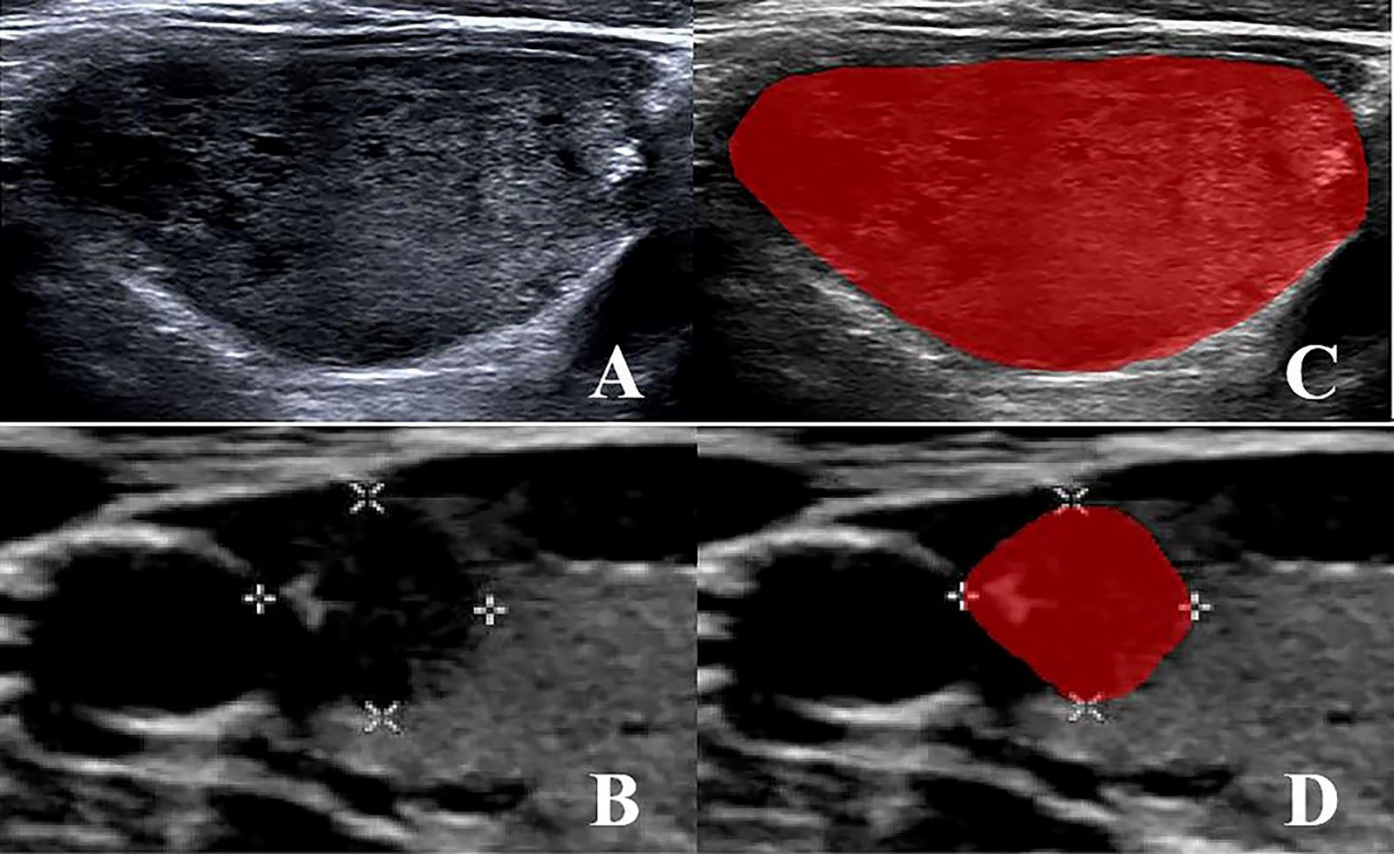
**(A, B)** MTC and PTC original 2D image; **(C, D)** MTC and PTC region of interest (ROI) segmentation image.

A 2D image of the largest cross-section tumor was selected for import into the ITK-SNAP software (version 4.0, www.itksnap.org). Initially, a senior radiologist with 10 years of experience (radiologist 1) outlined the edge of the lesion and identified the region of interest (ROI) as shown in **Figure 2**. After 4 weeks, 2D images of 30 randomly selected patients were outlined again by radiologist 1. In parallel, radiologist 2, with 15 years of experience, sketched the ROIs from 2D images of the above 30 patients as described above. Notably, neither radiologist was aware of the patient’s pathology to ensure an unbiased assessment. Additionally, we pre-processed the images before extracting the features because there are differences in the settings and acquisition parameters of ultrasound equipment from different brands, which can affect the final prediction results. This preprocessing included gray-scale normalization, discretization, and resampling of the region of interest (ROI).

#### 2.2.3 Radiomics feature extraction

The Pyradiomics module in Python 3.8.7 was utilized to extract features. First, the intraclass correlation coefficient (ICC) was computed to assess intra-observer and inter-observer consistency. Features with an ICC > 0.9 both within and between groups were retained. Next, any feature with zero variance was removed. Subsequently, the maximum correlation minimum redundancy algorithm (mRMR) was applied to select features with the highest correlation and the lowest redundancy. The variance inflation factor (VIF) was then calculated to evaluate the collinearity between the ultrasound features, and features with a correlation coefficient < 0.7 were selected. Finally, radiomics features with high robustness were obtained.

#### 2.2.4 Model building

Building machine learning models using the sklearn module in Python 3.8.7.

The clinical model was based on various parameters extracted from clinical baseline data, pathological features, and ultrasound characteristics. Clinical indicators whose differences were statistically significant in univariate analyses were included in multivariate logistic regression analyses to screen for independent predictors that differentiated between pathologic types of thyroid cancer in order to construct the clinical model.

Based on the selected optimal radiomic feature set, four radiomic models were constructed using Multilayer Perceptron (MLP), Support Vector Machine (SVM), Random Forest (RF), and XGBoost algorithms, respectively. Patients were divided into a training group and a validation group in a 7:3 ratio using stratified random sampling. To reduce overfitting, ten-fold cross-validation was performed on each model. In order to optimize the predictive performance of the models, a grid search method was used to fine-tune the model parameters.

ROC curves were plotted to compare the area under the curve (AUC) of the four radiomics models described above. The highest performing radiomics model was selected to be combined with clinical independent predictors to construct a comprehensive model.

#### 2.2.5 Statistical methods

Statistical analysis was performed using Python 3.8.7, R 4.2.2, and SPSS 26.0. Continuous variables were presented as mean ± standard deviation, while categorical variables were presented as frequencies with percentages. The comparison between the two groups was made using the two-independent sample t-test. The χ2 test was employed to compare categorical variables between the two groups.

The receiver operating characteristic (ROC) curve was utilized to evaluate the ability of the clinical model, radiomics model, and comprehensive model in distinguishing between MTC and PTC. Additionally, the area under the ROC curve (AUC), sensitivity, specificity, positive predictive value, negative predictive value and accuracy based on these models were calculated. The AUC values of different models were compared using the Delong test.

Calibration curves were applied to evaluate the performance of the models. Furthermore, clinical decision curves analysis (DCA) was utilized to assess the clinical utility of the three models.

A significance level of *P* < 0.05 was considered statistically significant.

## 3 Results

### 3.1 Comparison of baseline clinical, pathologic, and ultrasonographic features of MTC and PTC

**Table 1** shows that among 213 patients with thyroid cancer, there were statistically significant differences between MTC and PTC in terms of gender, age, clinical symptoms, past history of HT, and tumor TNM stage (all *P* < 0.05). The differences in family history of thyroid cancer, ETE and lymph node metastasis between the two groups were not statistically significant (all *P* > 0.05).

**Table 1:** Univariate analysis of baseline clinical and pathological characteristics (n).

Baseline information	MTC (n=82)	PTC (n=131)	χ^2^ */t*	*P*
**gender**			17.745	0.000
male	41 (50%)	29 (22.1%)		
female	41 (50%)	102 (77.9%)		
**age**	47.04 ± 12.94 (20~72)	41.9 ± 11.68 (15~74)	-2.925	0.004
**symptoms**			5.616	0.018
Yes	9 (11%)	3 (2.3%)		
None	73 (89%)	128 (97.7%)		
**family history of thyroid cancer**			0.000	0.998
Yes	5 (6.1%)	8 (6.1%)		
None	77 (93.9%)	123 (93.9%)		
**HT**			30.378	0.000
Yes	7 (8.5%)	58 (44.3%)		
None	75 (91.5%)	73 (55.7%)		
**ETE**			1.779	0.182
Yes	44 (53.7%)	58 (44.3%)		
None	38 (46.3%)	73 (55.7%)		
**LN metastasis**			0.066	0.798
Yes	48 (58.5%)	79 (60.3%)		
None	34 (41.5%)	52 (39.7%)		
**TNM staging**			23.270	0.000
T1N0M0	24 (29.3%)	44 (33.6%)		
T1N1M0	22 (26.8%)	55 (42.0%)		
T2N0M0	10 (12.2%)	8 (6.1%)		
T2N1M0	13 (15.9%)	23 (17.6%)		
T3N1M0	13 (15.9%)	1 (0.8%)		

As shown in **Table 2**, there were statistically significant differences between the ultrasound characteristics of MTC and PTC in terms of lesion size, composition, echogenicity, and margins (all *P <* 0.05). However, no statistically significant differences were observed in location, aspect ratio, calcification and blood flow (all *P* > 0.05)

**Table 2:** Univariate analysis of ultrasound characteristics (n).

Ultrasound sonogram	MTC (n=90)	PTC (n=135)	χ^2^ */t*	*P*
**size (cm)**	2.13 ± 1.49 (0.43-6.3)	1.52 ± 0.78 (0.45-4.21)	3.608	0.000
**location**			0.427	0.514
left	48 (53.3%)	66 (48.9%)		
right	42 (46.7%)	69 (51.1%)		
**margin**			35.371	0.000
smooth	31 (34.4%)	6 (4.4%)		
rough	59 (65.6%)	129 (95.6%)		
**echogenicity**			17.104	0.000
hypoecho	70 (77.8%)	68 (50.4%)		
extremely- hypoecho	20 (22.2%)	67 (49.6%)		
**calcification**			2.020	0.155
Yes	74 (82.2%)	120 (88.9%)		
None	16 (17.8%)	15 (11.1%)		
**composition**			4.823	0.028
solid	85 (94.4%)	134 (99.3%)		
cystic-solid	5 (5.6%)	1 (0.7%)		
**CDFI**			0.865	0.352
Yes	53 (58.9%)	71 (52.6%)		
None	37 (41.1%)	64 (47.4%)		
**Blood vessel distribution**			1.532	0.675
Type I	37 (41.1%)	64 (47.4%)		
Type II	6 (6.7%)	7 (5.2%)		
Type III	10 (11.1%)	10 (7.4%)		
Type IV	37 (41.4%)	54 (40%)		
**Adler grade**			1.881	0.597
0	37 (41.1%)	64 (47.4%)		
1	5 (5.6%)	6 (4.4%)		
2	5 (5.6%)	11 (8.1%)		
3	43 (47.8%)	54 (40%)		
**aspect ratio**			3.840	0.050
Yes	38 (42.2%)	75 (55.6%)		
No	52 (57.8%)	60 (44.4%)		

Data with *P* < 0.05 in univariate analysis were included in a multivariate logistic regression model to determine the independent factors affecting the differentiation between MTC and PTC. Ultimately, the study found that the patient’s gender, symptoms, TNM staging, previous history of HT, as well as the margin and echogenicity of the lesion were independent factors influencing the differentiation between the two (all *P* < 0.05), as shown in **Table 3**.

**Table 3:** Multivariate Logistic regression analysis of clinical, pathological, and ultrasound characteristics.

Variable	*B* value	standard error	Wald χ^2^	*P*	OR (95%*CI*)
**gender**	1.830	0.417	19.456	0.000	6.289 (2.778-14.239)
**age**	0.017	0.017	1.070	0.301	1.017 (0.985-1.051)
**symptoms**	-2.194	0.981	5.002	0.025	0.111 (0.016-0.762)
**HT**	2.209	0.557	15.712	0.000	9.108 (3.055-27.152)
**TNM staging**			6.140	0.029	
**TNM staging (1)**	-4.821	2.216	4.734	0.030	0.008 (0.000-0.620)
**TNM staging (2)**	-5.043	2.152	5.491	0.019	0.006 (0.000-0.438)
**TNM staging (3)**	-4.102	1.847	4.933	0.026	0.017 (0.000-0.618)
**TNM staging (4)**	-3.760	1.830	4.223	0.040	0.023 (0.001-0.840)
**size**	-0.550	0.431	1.624	0.203	0.577 (0.248-1.344)
**composition**	1.190	2.040	0.340	0.560	3.287 (0.060-179.365)
**margin**	-2.245	0.686	10.709	0.001	0.106 (0.028-0.406)
**echogenicity**	1.239	0.413	9.012	0.003	3.453 (1.538-7.755)

### 3.2 Radiomics feature selection

**Figure 3:**
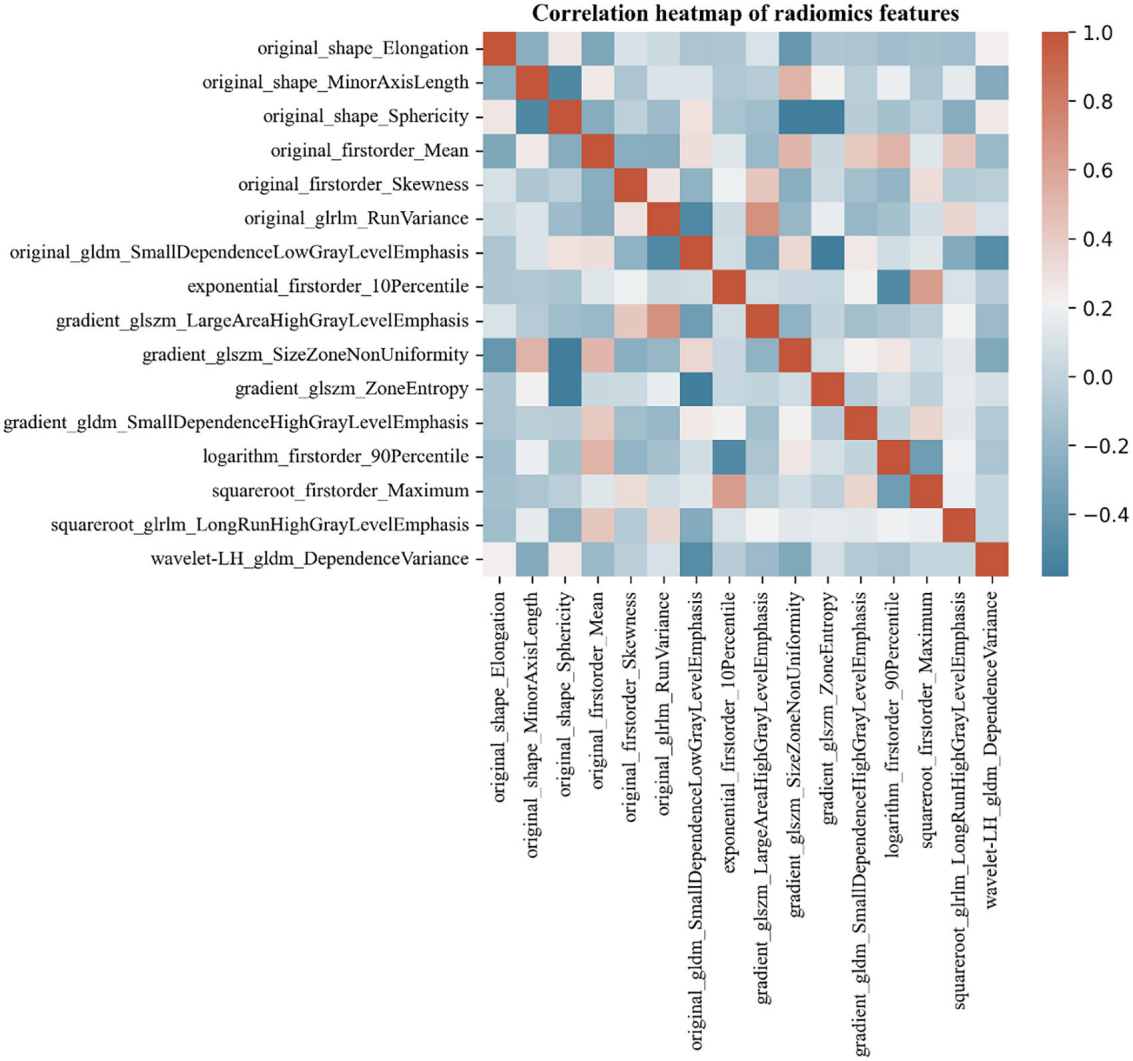
Correlation heat map of MTC and PTC ultrasound-radiomics features. Color indicates correlation, and the darker the color, the higher the correlation. Red indicates a positive correlation and blue indicates a negative correlation.

The Pyradiomics software package was utilized to select 2D images of the maximum tumor cross-section, from which a total of 873 features were extracted. After performing dimensionality reduction using regression, 16 radiomics features were retained. These features consist of 5 first-order features (Firstorder), 3 Shape features (Shape), 2 gray level run length matrices (GLRLM), 3 gray level size zone matrices (GLSZM), and 3 gray level dependence matrices (GLDM). The Spearman correlation heatmap of each radiomics feature was depicted in **Figure 3**.

### 3.3 Modeling and predicting diagnostic efficacy

**Figure 4:**
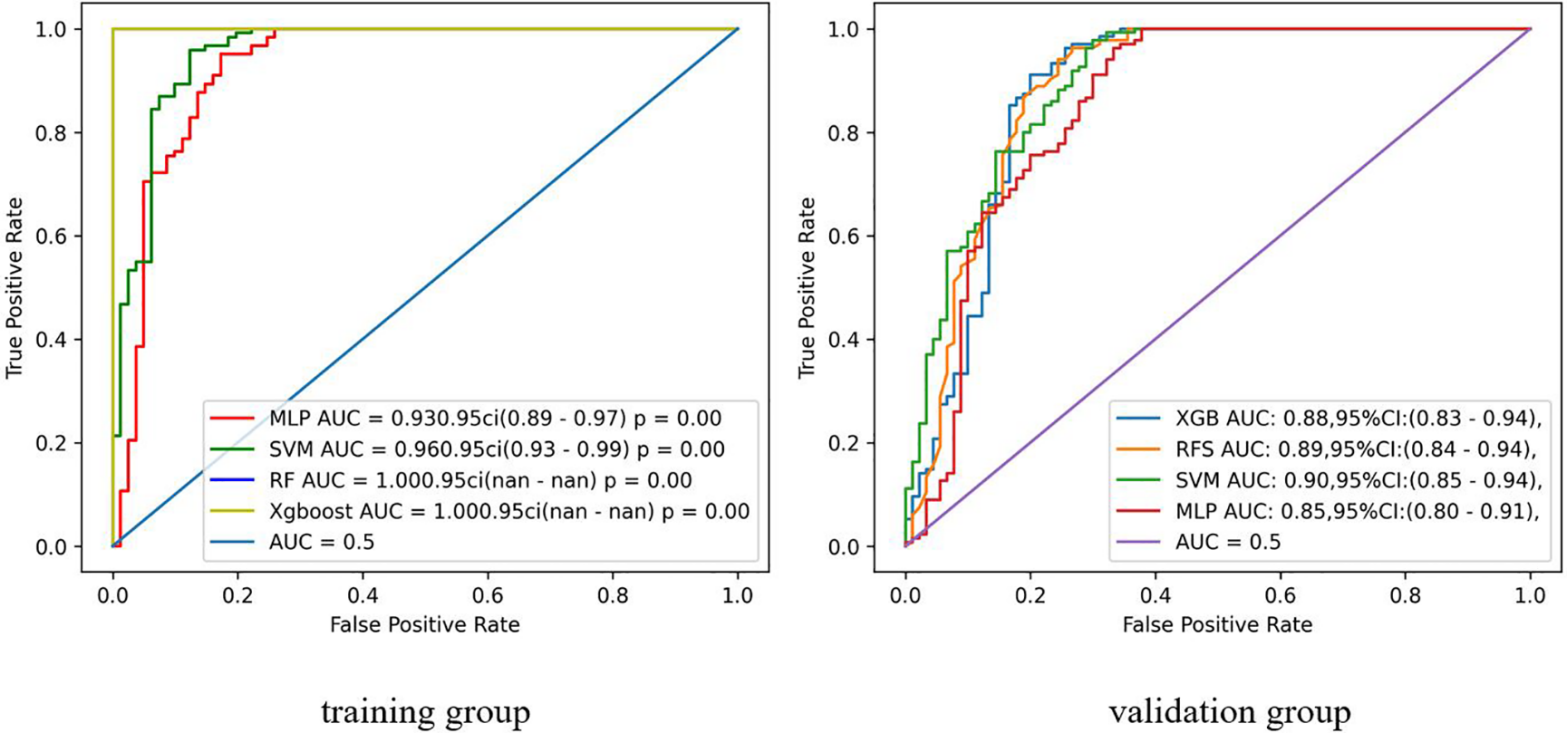
ROC curve and area under the curve (AUC) of four machine learning models.

**Figure 4** shows the ROC curves for the radiomics models constructed using the four machine learning algorithms. In total, we used four machine learning algorithms to develop radiomics models. In the training set, the RF and XGboost algorithm models exhibited overfitting with an AUC of 1.000, making these two algorithm models unsuitable for continued use in this study. Compared to the MLP algorithmic model, the SVM algorithmic model showed a higher AUC in the training set, with an AUC of 0.96 and 95% CI of 0.93-0.99. Therefore, the SVM algorithm model was ultimately selected as the radiomics model for the validation set, and we proceeded to the next phase of the study to construct a comprehensive model.

Ultimately, three models, namely a clinical model, a radiomics model, and a comprehensive model were constructed based on baseline clinical, pathologic, ultrasonographic data and radiomics features. The final test results of these models are shown in **Table 4**.

**Table 4:** The clinical effectiveness of three models in differentiating MTC from PTC.

	AUC	95%*CI*	Sensitivity	Specificity	Positive predictive value	Negative predictive value	Accuracy
**Clinical model**	0.83	0.78-0.89	0.85	0.67	0.64	0.87	77.8%
**Radiomics model**	0.90	0.85-0.94	0.93	0.71	0.69	0.96	84.4%
**Comprehensive model**	0.93	0.90-0.97	0.88	0.82	0.77	0.91	85.8%

**Figure 5:**
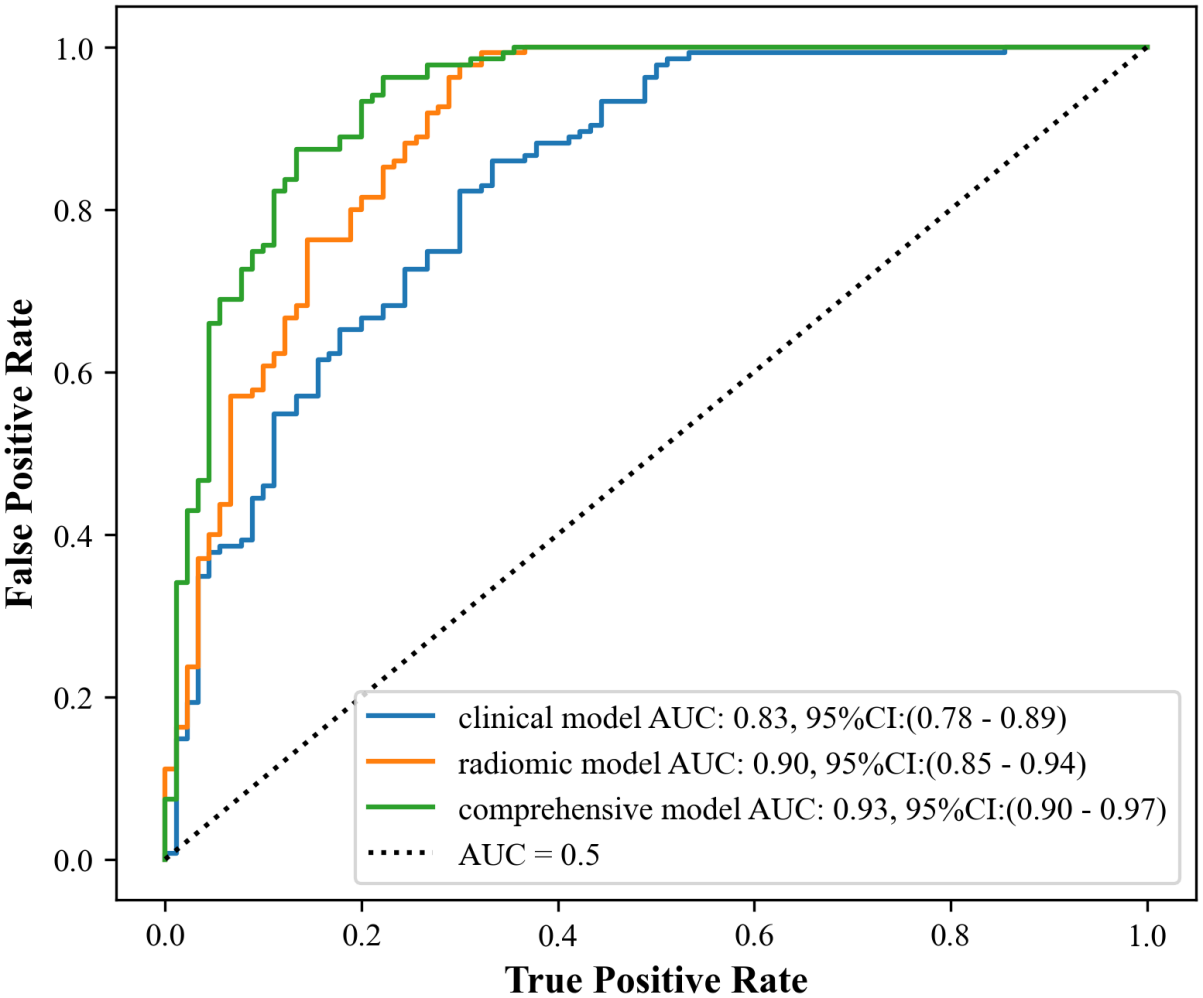
ROC curve and area under the curve (AUC) of the three models.

**Figure 5** illustrates the performance of the three models. The results of the Delong test indicated that the comprehensive model had a significant advantage in diagnostic performance, with a larger area under the ROC curve compared to both the radiomics model (Z = -2.017, *P* = 0.044) and the clinical model (Z = -3.719, *P* < 0.001), and these differences were statistically significant. However, there was no statistically significant difference in the area under the ROC curve when comparing the radiomics model to the clinical model (Z = -1.712, *P* = 0.087).

**Figure 6:**
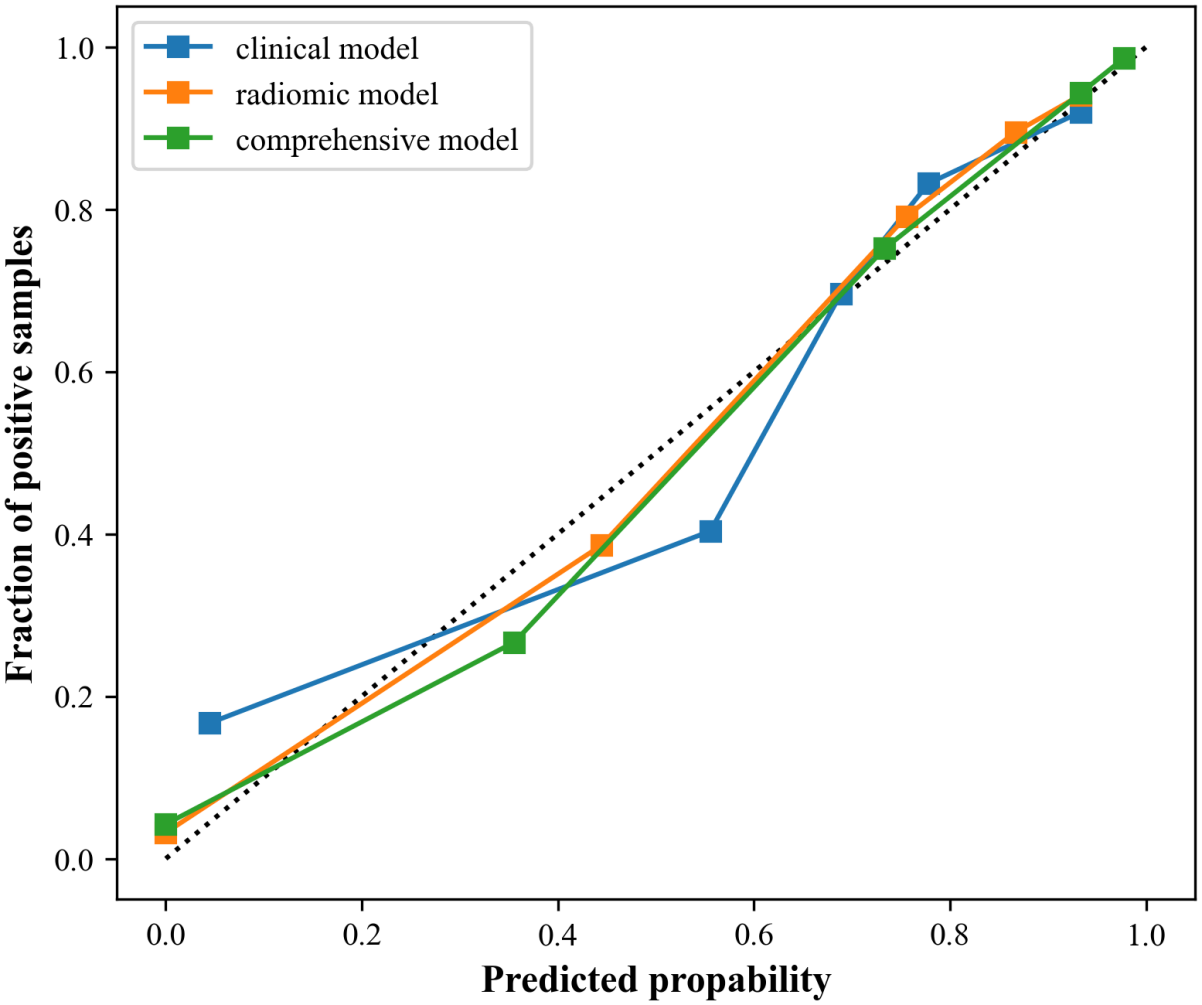
Calibration curves of the three models. The closer to the dotted line, the better the stability of the model.

**Figure 7:**
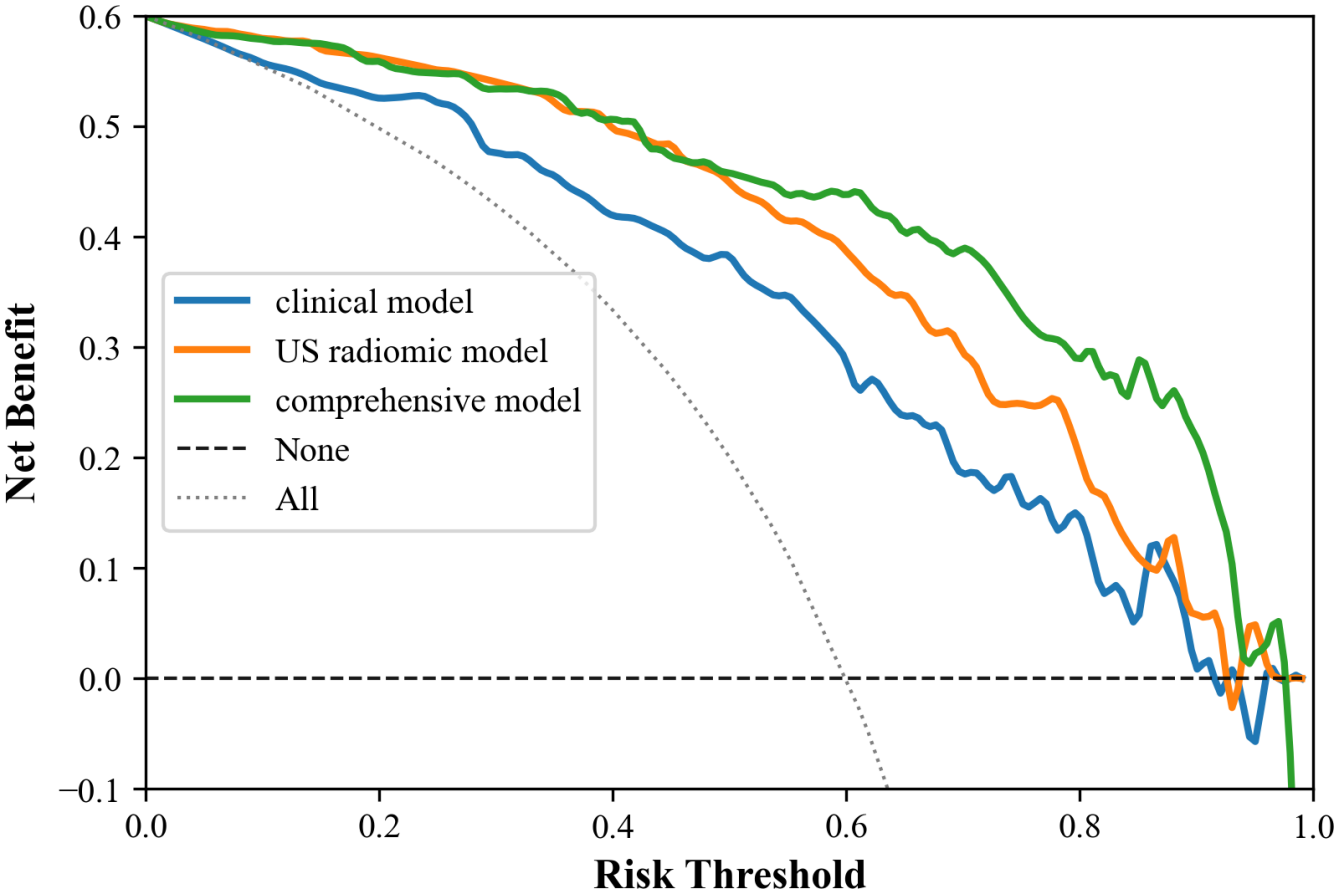
Decision curves of the three models. Within the threshold range of 0.0 to 1.0, the comprehensive mode has the best clinical net benefit, followed by the omics model, and the clinical model is the worst.

In addition, the calibration curves and decision curves for the three models are shown in **Figures 6** and **7**, respectively.

## 4 Discussion

MTC, a malignant neuroendocrine tumor, has a much lower incidence compared to PTC. Over the years, there has been rapid progress in both basic and clinical research on MTC, leading to a significant increase in our understanding of the disease (18, 19). Ultrasound is considered the most valuable imaging modality for assessing the risk of malignancy in thyroid nodules. However, some MTC and PTC cases share significant similarities in ultrasound imaging presentations. This limitation can be overcome by leveraging radiomics, which allows for the quantitative extraction of features from medical images, enabling the identification of features that are not discernible to the naked eye.

By accurately distinguishing between MTC and PTC before surgery, radiomics can greatly assist clinicians in selecting the optimal treatment plan, thereby promoting individualized and targeted treatment strategies (20, 21).

In this study, we constructed a clinical model based on patients’ clinical baseline data, pathological information and ultrasonic features to predict the pathologic type of thyroid cancer before surgery. The sensitivity and accuracy of this model were found to be 0.85 and 77.8%, respectively. The findings suggested that patient gender, symptoms, past history of HT, TNM staging, lesion margins and echogenicity are pivotal in distinguishing MTC from PTC.

Studies have shown that the incidence of MTC was relatively comparable in males and females, while PTC was more prevalent in females (*P* < 0.001). Certain studies have demonstrated that a greater proportion of sporadic MTC patients are female, whereas familial MTC appears to be more frequently inherited in male patients (19). Numerous studies have proven a direct correlation between the expression of estrogen receptor alpha (ERα) in females and the occurrence of MTC, thus explaining the elevated incidence of PTC in the female population (22).

Additionally, in contrast to PTC, MTC is often associated with distressing symptoms such as hoarseness, neck pain, or neck masses, which is consistent with previous research (23).

We found that patients with PTC were more likely to have a past history of HT (*P* < 0.001). Several studies have demonstrated that patients with PTC have a higher risk of coexisting with autoimmune thyroid disorders (especially HT) (24). HT patients often have elevated serum levels of thyroglobulin antibodies (TgAb) and thyroid peroxidase antibodies (TPOAb). These antibodies not only induce immune-mediated damage to the thyroid tissue, but may also promote the progression and metastasis of PTC. Furthermore, studies have indicated that heightened levels of TPOAb and TgAb may be key factors influencing the onset and progression of PTC (24).

We also found that the TNM staging of MTC patients was generally higher than that of PTC patients (all *P<* 0.05). This may be related to factors such as the biological characteristics, disease progression, and timing of diagnosis and treatment of the two thyroid cancers (25). In general, MTC is more aggressive and malignant, and may progress faster than PTC, all of which may contribute to higher TNM staging for MTC patients.

In our study, 34.4% of MTC cases exhibited the characteristic of smooth edges, a proportion significantly higher than the 4.4% observed in PTC cases, and this observation is consistent with the findings of other studies (26). These findings suggest that MTC may present ultrasound features similar to those of benign thyroid nodules.

Hypoechoic appearance is a common ultrasonic feature of thyroid malignancies (27). In our study, all lesions were hypoechoic or extremely hypoechoic, while PTC was more likely to be extremely hypoechoic (lower than the anterior cervical muscle). This distinction in echo patterns may serve as one of the characteristics for differentiating PTC from MTC.

However, in other aspects, MTC did not show significant differences compared to PTC, indicating that there is some overlap in the sonogram performance of the two pathologic types.

Tumor inhomogeneity in conventional ultrasound sonograms can potentially indicate intra-tumor heterogeneity, but this feature is challenging for human eyes to discern. Therefore, this study employs machine learning techniques to identify images, screen features, and express the heterogeneity within the tumor, subsequently predicting the pathological types of thyroid nodule (28).

In this study, a total of 873 radiomics features were extracted by selecting the 2D images of the largest cross-section of the tumor, and 16 highly robust features were retained after dimensionality reduction. These features included 5 first-order features, 3 shape features, 2 GLRLM features, 3 GLSZM features, and 3 GLDM features. The first-order features are based on the pixel gray distribution of the original image and the image after various filters. The related feature had original first order, original first order skewness, exponential_firstorder_10Percentile, logarithm_firstorder_90Percentile, and square root first-order maximum. The results of this study suggest that PTC was more widely distributed than MTC, and the asymmetry was more significant.

Shape features mainly describe the shape of the lesion and its similarity to sphericity. The relevant features of this study were original shape elongation, original shape minor axis length, and original shape sphericity. The results showed that PTC had a more irregular morphology and larger elongation compared to MTC. This may be related to the characteristics of PTC. Smaller PTC often displays morphological characteristics with aspect ratios > 1. With the growth of PTC, its anterior and posterior diameters are limited by the thyroid capsule, but it can grow in other directions, including outward growth. As a result, PTC had a more irregular morphology than MTC.

Texture features are visual features that reflect homogeneity in images. Although both MTC and PTC are malignant tumors, they have different origins, compositions, internal roughness and gray distributions of nodules. However, these differences are not easily detected by the naked eye and can be identified through texture features, which are not influenced by subjective factors (29). This study selected eight texture characteristics, including gradient GLSZM large area high gray level emphasis, gradient GLSZM size zone non-uniformity, gradient GLSZM zone entropy, original GLDM small dependence low gray level emphasis, gradient GLDM small dependence high gray level emphasis, wavelet – LH GLDM dependence variance, original GLRLM run variance, and square root GLRLM long run high gray level emphasis. In this study, the number of texture features was the largest, indicating that the internal structure and heterogeneity of the tumor were closely related to the pathological classification of nodules, and PTC exhibited rougher texture and higher heterogeneity than MTC (30, 31).

In this study, we chose the ten-fold cross-validation method, which divides the dataset into ten subsets and ensures that each subset has the opportunity to be used as both a training set and a test set, resulting in more reliable model performance. This rigorous validation approach helped minimize the variance of performance estimates and provided a comprehensive understanding of the model’s ability to generalize over unseen data, thereby reducing the risk of overfitting.

Additionally, we utilized four machine learning algorithms to construct the radiomics model, and ultimately selected SVM as the optimal classifier for inclusion in the experiment. SVM classifier demonstrates numerous unique advantages in handling small sample sizes, nonlinear patterns, and high-dimensional pattern recognition, making it the optimal choice for preoperative identification of MTC and PTC.

The results of the present study demonstrated that the comprehensive model had higher diagnostic validity compared with the clinical and radiomics models, which is consistent with previous findings (32–34). The design of the comprehensive model capitalizes on the strengths of the two separate models, resulting in higher specificity, negative predictive value, accuracy, and AUC than the separate models. Statistically significant differences were noted when comparing the comprehensive model with the clinical model (Z=-3.791, *P*<0.001) and the radiomics model (Z=-2.017, *P*=0.044). However, no statistically significant difference was found when comparing the clinical model with the radiomics model (Z=-1.712, *P*=0.087).

Moreover, calibration curves demonstrated that the comprehensive model and the radiomics model exhibited better stability than the clinical model. DCA results revealed that the comprehensive model provided greater net benefit in preoperative prediction of thyroid nodule pathology type within the threshold range of 0.0 to 1.0, and its advantages were more pronounced.

Recently, some researchers have proposed that machine learning-based lateral cervical lymph node metastasis (LLNM) prediction models have demonstrated a good prediction performance in MTC patients (35). This study used a larger sample size and combined clinical and ultrasound data, demonstrated a good discriminatory ability in predicting LLNM in patients with MTC, especially in predicting occult LLNM (35). Another investigator developed and validated a predictive model capable of predicting the risk of distant metastasis (DM) early after MTC (36). Patient age, surgical approach, T-stage and N-stage were found to be independent risk factors that are associated with the risk of early DM after MTC, and the developed prediction model demonstrated good discriminatory ability in the prediction of the risk of early DM after MTC, and was able to efficiently screen out high-risk patients (36). However, the machine models constructed in these two studies only incorporated textual information from patients’ clinical baseline, pathology, and ultrasound image data, and did not perform machine learning tasks such as reading and analyzing image images for MTC and extracting image histology features.

There are some limitations to this study: (1) This was a single-agency retrospective study, which may introduce bias in the results. A prospective multi-center study could be conducted to further validate the stability of the findings. (2) The images used in this study were obtained from three different ultrasound instruments of various brands and models, which may lead to image heterogeneity. (3) Because the possibility of MTC was not considered preoperatively in some patients, these cases did not undergo preoperative serum calcitonin test. The aim of this study was to investigate the difference between PTC and MTC without considering the effect of serum calcitonin. Moreover, there were relatively few MTC cases, and no stratified analysis based on nodule size was performed in this study. Future studies will collect more cases, incorporate preoperative serum calcitonin indicators into clinical models, and stratify analysis based on nodule size.

## 5 Conclusion

Radiomics allows for the quantitative analysis of tumors by extracting high dimensional features from images that are difficult to discern with the naked eye, thereby paving the way for accurate cancer diagnosis. In this study, we successfully developed and validated a radiomics model to differentiate MTC from PTC, providing a more objective and informative approach to reflect the internal heterogeneity of the lesion. This advancement is critical in assisting physicians in making optimal management decisions in the era of personalized medicine.

## Data Availability

The original contributions presented in the study are included in the article/supplementary material. Further inquiries can be directed to the corresponding author.
